# Scoping review: the empowerment of Alzheimer’s Disease caregivers with mHealth applications

**DOI:** 10.1038/s41746-021-00506-4

**Published:** 2021-09-07

**Authors:** Eunhee Kim, Andrius Baskys, Anandi V. Law, Moom R. Roosan, Yan Li, Don Roosan

**Affiliations:** 1 Western University of Health Sciences, College of Pharmacy, Department of Pharmacy Practice and Administration, Pomona, CA USA; 2 Western University of Health Sciences, College of Graduate Biomedical Sciences, Pomona, CA USA; 3 Chapman University, School of Pharmacy, Department of Pharmacy Practice, Pomona, CA USA; 4 Claremont Graduate University, School of Information Systems & Technology, Pomona, CA USA

**Keywords:** Alzheimer's disease, Quality of life

## Abstract

Alzheimer’s Disease (AD) is one of the most prevalent neurodegenerative chronic diseases. As it progresses, patients become increasingly dependent, and their caregivers are burdened with the increasing demand for managing their care. Mobile health (mHealth) technology, such as smartphone applications, can support the need of these caregivers. This paper examines the published academic literature of mHealth applications that support the caregivers of AD patients. Following the PRISMA for scoping reviews, we searched published literature in five electronic databases between January 2014 and January 2021. Twelve articles were included in the final review. Six themes emerged based on the functionalities provided by the reviewed applications for caregivers. They are tracking, task management, monitoring, caregiver mental support, education, and caregiver communication platform. The review revealed that mHealth applications for AD patients’ caregivers are inadequate. There is an opportunity for industry, government, and academia to fill the unmet need of these caregiver.

## Introduction

The global population is aging at a rapid pace. With advancements in medicine and public health, one in 11 people globally was over 65 in 2019. According to the United Nations, the number is projected to reach one in six people by 2050^1^. In the United States, the population of Americans over the age of 65 is projected to be 83.7 million in 2050, almost double the 43.1 million in 2012 according to the U.S. Census Bureau^2^. With the changing population demographic, an increase in healthcare costs is inevitable. Healthcare expenditure per capita for the population of Americans 65 and older increased by over 50% from 1996 to 2016^3^. Chronic conditions are primarily responsible for the higher healthcare cost, and it is estimated that ~171 million Americans will have at least one chronic illness by 2030^4^. Among those, Alzheimer’s Disease (AD) is one of the most prevalent in the elderly population. It is estimated that people with clinical AD would reach 9.3 million by 2060^5^.

Despite the growing number of AD in the elderly population, there is no cure for AD yet. Current treatment options mainly focus on slowing down the disease progression and helping to maintain cognitive function. For the pharmacologic treatment of AD, cholinesterase inhibitors, such as donepezil, and an *N*-methyl-d-aspartate (NMDA) receptor antagonist, memantine is used. Majority of drugs approved by the U.S. Food and Drug Administration (FDA) support treatment only if the patients are in the early stages of AD. For nonpharmacologic treatments, exercise programs and cognitive rehabilitation are used to promote rehabilitation^6,7^. Thus, early detection and diagnosis of AD can be significantly beneficial for treating the patients^8^.

Owing to the neurodegenerative nature of AD, Alzheimer’s patients need an increasing amount of care as the disease progresses^9^. Often, family members become the caregivers for the patients. More than 15 million Americans provided care of AD and other dementia patients with ~18.1 billion hours of care in 2015 alone^9^. Early diagnosis of AD is also crucial for caregivers, as it can give them more time to learn about the disease and become an integral part of planning care for the patient^10^.

Caregivers for AD patients often endure an enormous financial, emotional, and physical burden. According to a recent study, 69% of caregivers of AD patients felt a medium burden and the patient’s severity of the disease state was a factor significantly affecting them^11^. When compared with non-caregivers, caregivers were more likely to be depressed and also utilized more negative than positive coping strategies^12^. Giving support to caregivers is as important as providing direct care to AD patients because the well-being of caregivers is a direct indicator of the care quality they provide^10^.

Caregivers need education, social support, and effective strategies to maximize the quality of care given while maintaining their own wellbeing. Interventions that can make caregiving easier for the caregivers include education, counseling, support groups, and case management support^9^. The core of caregiving lies in understanding the disease and knowing how the patients will change through the disease progression. With proper and accessible diagnosis tools, caregivers, often one of the closest people to the patients, can identify alerting factors that aid the early detection of AD.

Mobile health (mHealth) applications are health-related smartphone applications that offer functionalities to improve patient health^13–15^. mHealth applications can be designed to provide accessible tools to support caregivers, including early AD diagnosis. Many mHealth applications are available for AD patients. Most of these applications are designed for patients to self-manage AD states. For example, Backup Memory is an application that helps AD patients remember people by saving images of people who are known to them^16^, and Test Memory Game is an application that provides memory exercise games with shapes, numbers, and letters for AD patients^16^. Very few applications are available to assist AD caregivers. The National Institute on Aging (NIA) is currently funding 123 clinical trials supporting assistive devices and technology for AD and 84 trials supporting care and caregiver interventions. However, no projects explored mobile applications for supporting caregivers including diagnostic tools^17^. Thus, there is a gap in the literature to design and develop mHealth applications specifically for caregivers of AD patients. The objective of this scoping review is to examine the published academic literature of mHealth applications that support the caregivers of Alzheimer’s patients.

## Materials and methods

### Data sources and search strategy

**Fig. 1:**
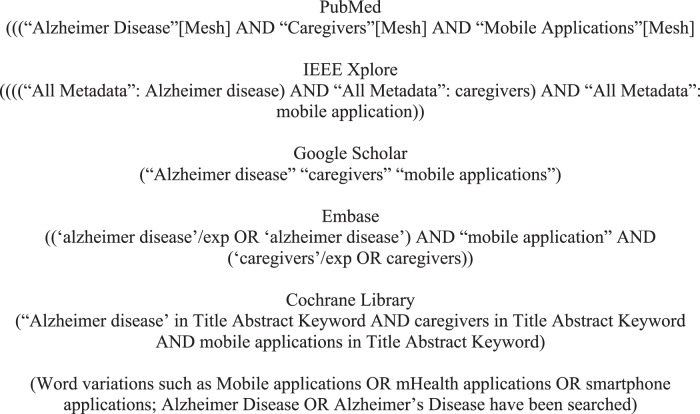
Database search queries. This figure shows the keywords used in each electronic database for the literature search for mobile applications for caregivers of Alzheimer’s patients.

**Fig. 2:**
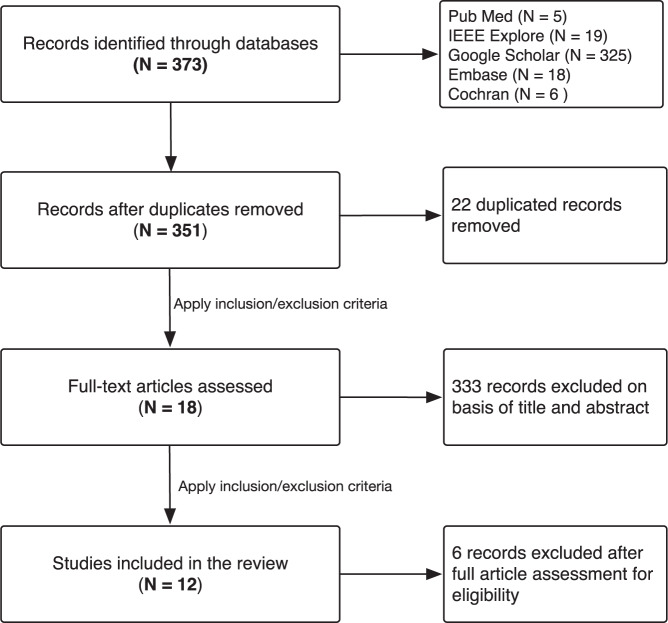
The flowchart of the search for eligible studies. This flowchart shows the steps of how the 12 articles for the review of mobile applications for caregivers of Alzheimer’s patients were extracted from five electronic databases incorporating the inclusion and exclusion criteria.

We followed the Preferred Reporting Items for Systematic reviews and Meta-Analyses extension for Scoping Reviews (PRISMA-ScR) to guide the literature search. We searched published literature in English in five electronic databases, including PubMed, Embase, Cochrane Library, Google Scholar, and IEEE Explore, between January 2014 and September 2020. The date was extended to 22 January 2021 in the most recent research. The databases were searched with combinations of three keywords “Alzheimer disease,” AND “caregivers,” AND “mobile applications” and their synonyms (see Fig. 1). An initial screening removed all duplicates. Then we examined the title and abstract of these non-duplicated articles based on the inclusion and exclusion criteria (see Table 1). Finally, full-text reviews were performed on the remaining articles and a total of 12 studies were included in our final review (see Fig. 2). Two researchers independently performed data charting.

**Table 1:** Inclusion and exclusion criteria for articles included in the final review.

Exclusion criteria	Inclusion criteria
Articles published between 1 January 2014 and 22 January 2020	Articles not available in full text
Articles available in full text	Articles not published in English
Articles published in English	Review papers
Articles addressing caregivers of Alzheimer’s patients	Articles including digital tools but not mobile applications
Articles addressing mobile applications specifically as support tools for caregivers	Articles not addressing caregivers of Alzheimer’s patients
Articles addressing the contents of mobile applications that facilitate caregiving	Articles focusing on digital tools to support the care recipients and not the caregivers
	Articles not specifying the contents of mobile applications that facilitate caregiving

### Data extraction and analysis

The review of the titles and abstracts of the non-duplicated articles identified potential articles that meet the inclusion and exclusion criteria. Full-text articles were then reviewed to extract data about the mHealth applications, specifically the platform system, the objectivity of the application (see Table 2), and application specifications for caregiver support. After the application specifications were extracted, six themes emerged based on the types of support provided by these applications (see Table 3). Two independent reviewers performed the extraction and grouping, and a third reviewer verified the work. All results were cross-examined and checked for accuracy. If there were any conflicts in reaching a consensus for any articles, a third reviewer resolved conflicts.

**Table 2:** Summary of the 12 articles extracted.

Article name	Author name	Application name	Application platform system	Application objectives
The potential of information technology to navigate caregiving systems: perspectives from dementia caregivers	Ruggiano et al.^29^	*Care IT*	Android	Meet the common education and support needs of Alzheimer’s disease and related dementias (AD/RD) caregivers
Environment-aware system for Alzheimer’s patients	Barreto et al.^18^	*AlzSense*	Android	Provide the caregiver with the possibility of accessing the patient’s information on the server
*iCare*: applying IoT technology for monitoring Alzheimer’s patients	Aljehani et al.^19^	*iCare*	IOS	Facilitate Alzheimer’s caregiving and avoid burnout
A mobile cloud-based system for Alzheimer’s disease	A. Ghanem, H. Alkhal^20^	N/A One application for the caregiver and one application for the patient	IOS	Create two separate applications for Alzheimer’s patients and the caregiver to assist with task management, patient tracking, and setting up answers to patient’s commonly asked questions
A pervasive and ubiquitous mobile health application for tracking people with disabilities	Vergara et al.^21^	*Acompáñame*	Android	Help caregivers to track people with disabilities
*CareD*: non-pharmacological assistance for dementia patients	Siddiq et al.^22^	*CareD*	Android	Improve the quality of life of dementia patients and facilitate their caregivers by providing non-drug tool that provides a single platform that merges activities and cognitive therapy sessions for AD patients
*UnderstAID*, an ICT platform to help informal caregivers of people with dementia: a pilot randomized controlled study	Núñez-Naveira et al.^27^	*UnderstAID*	Android and IOS	Utilize information and communication technology to support informal caregivers of people with dementia, especially when they need to cope with their feelings of overburden or isolation
Designing the *ReACT* App to support self-management of people with dementia: an iterative user-involving process	Øksnebjerg et al.^28^	*ReACT*	IOS	Support self-management needs of people with dementia
Analyzing and implementing a mobile reminder system for Alzheimer’s patients	Alharbi et al.^23^	*MemoryLane*	Android	Give Alzheimer’s patients the ability to have small memory that can help them remember all tasks to live
Mobile health applications and android toolkit for Alzheimer’s patients, caregivers, and doctors	Gupta et al.^24^	*AlzCare*	Android	Utilize smartphones to help patients in carrying out routine activities and assist caregivers to take proper care of the patient
SMAI - mobile system for elderly monitoring	Stutzel et al.^25^	*SMAI Caregiver*	Android	Make the communication between caregiver and health team faster, facilitate the care and elderly support in their daily living activities, and provide the health team information about the patient’s condition more often and in an organized manner
BLE Bluetooth Beacon based solution to monitor egress of Alzheimer’s disease sufferers from indoors	D. Surendran, M. Rohinia^26^	N/A	Android	Detect and notify caregivers about the departure of people to risk locations from their residence zone

*AD* Alzheimer’s disease, *RD* related dementias.

**Table 3:** Summary of themes based on the functionalities provided by the mobile applications to support caregivers.

Table 3	Care IT	AlzSense	iCare	A. Ghanem	Acompáñame	CareD	UnderstAID	ReACT	MemoryLane	AlzCare	SMAI Caregiver	D. Surendran
Tracking		✓	✓	✓	✓	✓			✓	✓	✓	✓
Task management			✓	✓		✓	✓	✓	✓	✓	✓	
Monitoring												
* Patient activity*		✓										✓
* Patient environment*		✓										
* Patient health parameters*	✓		✓								✓	
Caregiver mental support	✓											
Education	✓						✓			✓		
Caregiver communication platform	✓						✓					

## Results

A total of 351 non-duplicate articles were collected from the initial search, of which 18 articles were eligible for full-text screening based on title and abstract. After the full-text screening, 12 articles were included for the final review. No additional articles were identified from reviewing the references of relevant articles. Table 2 provides a summary of articles included in this scoping review.

The review identified six themes (see Table 3) based on the types of caregiving support functionalities that the applications provided for caregivers of AD patients. The six themes are tracking, task management, monitoring, caregiver mental support, education, and caregiver communication platform. Some functionalities were explicitly designed for the caregivers themselves through education and mental support, whereas some provided caregiving support through better patient management, such as patient monitoring. Many applications included overlapping functions. Details of each theme are discussed below.

### Tracking

Tracking of the AD patient was featured in 9 out of the 12 (75%) applications^18–26^. The most identifiable symptom of AD is forgetfulness and memory loss. AD patients are at risk of getting lost and not being able to find their way back home. Eight applications track the patient’s current location, allowing caregivers to check at any given time^19–26^. Only one application, *AlzSense*, offered tracking by keeping a history of the patient’s location changes by date and time^18^. In addition, three applications offered an alert system for caregivers. For example, the application by A. Ghanem et al.^20^ provided a function where the patient can enable an emergency alert system to notify the caregiver of their location. With *Acompáñame*^21^, the caregiver can define a geographical zone that is deemed safe for the patient to move about in, and the application will alert the caregiver when the patient goes outside the defined zone. *Acompáñame* also can help locate nearby places such as a hospital in case of an emergency^21^.

### Task management

Features that help task management of caregivers were included in 8 out of 12 (66.7%) applications^19,20,22–25,27,28^. As AD progresses and the patient becomes increasingly dependent on the caregiver to carry out daily activities, the caregiver is burdened with essential tasks for two people. Six applications allowed the caregivers to add daily tasks from appointments to the medication schedule^19,23–25,27,28^. These applications would send an automatic task reminder alert to the caregiver. The other two applications, *CareD* and the application by A. Ghanem et al.,^20,22^ require the caregivers to set up reminders for the patients. For example, the caregivers would set up timed alerts for when the patient should take medications or perform a daily task. Such a feature is beneficial for more independent AD patients in the earlier stages of the disease.

### Monitoring

The monitoring of care recipients was featured in five out of 12 (41.7%) applications^18,19,25,26,29^. This functionality can be further classified into three subcategories—patient activity, patient environment, and patient health parameters. Four applications offered just one of the monitoring categories, whereas *AlzSense*^18^ offered two different monitoring categories. For monitoring patient activity, *AlzSense*^18^ tracks the patient’s movement (e.g., falls) and sends the updated information to the caregiver, whereas the application by D. Surendran et al.^26^ tracks the patient’s movement that can be analyzed for patterns. Only one application, AlzSense^18^, tracks the patient’s environment temperature and humidity and sends the information to the caregiver. Three applications that track patient health parameters, including *SMAI Caregiver*, *iCare*, and *Care IT* met the criteria. With *SMAI Caregiver*^25^, caregivers can record the patient’s blood pressure and blood sugar readings on the application. Caregivers can also keep a record of the patient’s behavior, eating routine, and urination. With *iCare*^19^, the application monitors the patient’s heart rate that the caregivers can view at any time, *Care IT*^29^ allows caregivers to monitor and record the changes in the patient’s symptoms. All these monitoring parameters can be shared with the patient’s providers for better management of AD care.

### Caregiver mental support

Our review only identified one (8.3%) application, *Care IT*^29^, that provides mental health support for caregivers through the self-assessment of depression and burden using validated measures. The study, however, did not specify what valid measures to assess depression were used. Understanding the state of one’s mental health and the amount of burden can accurately prompt the caregiver to seek medical attention if needed.

### Education

Three out of the 12 (25%) applications, *Care IT*, *UnderstAID*, and *AlzCare*, featured an education section within the applications^24,27,29^. *Care IT* has links to educational resources about AD that caregivers can utilize^29^. *UnderstAID* has five modules with 15 different topics that can be beneficial for caregivers. The modules are as follows: Cognitive Declines, Daily Tasks, Behavioral Changes, Social Activities, and You as a Caregiver. These modules cover topics for caregiving as well as caring for oneself as a caregiver. They provide videos and images in addition to text and offer links to other websites for more information^27^. Finally, *AlzCare* has a Learning & Care Giving section in the application that contains basic information about AD diagnosis and caregiving. The AD diagnosis information can help caregivers accurately identify the patient’s current disease stage and learn how to handle the patient appropriately. However, the article does not specify what type of diagnosis information is available through the application. *AlzCare* also has a news section that the caregivers can get up-to-date information regarding care^24^.

### Caregiver communication platform

There were two applications (16.7%) that provided communication platforms for the caregivers. *UnderstAID*^27^ featured a Social Network section where caregivers can communicate with other caregivers to exchange information and opinions. The purpose of the communication platform was to allow caregivers to share their knowledge and opinions about caregiving with other caregivers^27^. This supports the need for caregivers to interact with other caregivers in similar situations, creating an outlet for additional mental support. *Care IT*^29^ provides a secure platform for sending patient-related information to the patient’s physicians. Changes in the patient’s symptoms tracked by the *Care IT* application can thus be easily communicated with the physicians. Other applications can adopt a similar platform to expand the caregiver community and network of support for caregivers.

In addition to the six themes discussed, several mHealth applications offered unique functionalities for the caregivers. The application by A. Ghanem et al.^20^ allows caregivers to add a list of relatives and friends that are close to the patient, including their names, photos, and phone number. Patients can access the list and make phone calls through the application. *UnderstAID* provides an option for the caregivers to fill out a customized questionnaire about the severity of the dementia of the AD patient, as well as the caregiver’s preferences, energy, and time availability for learning^27^. Such information is important to display personalized information to the caregivers.

As seen from the discussed themes, there were multiple types of caregiving support offered by these applications. However, none of them supported the incorporation of caregivers into patient care management. There were no applications that offered diagnostic tools for the caregivers to utilize. Although *Care IT*^29^ has a communication platform to send patient information to physicians, the application does not support diagnostic tools that the caregivers can use and communicate the results with physicians.

## Discussion

The goal of the study was to examine mHealth applications that support caregivers of AD patients. Following the PRISMA for scoping reviews, we reviewed 12 published literature of mHealth applications that support caregivers of AD patients and identified six themes of functionalities that provide both caregiving and emotional support for caregivers.

The most common support features (75%) in these applications were tracking, task management, and patient monitoring. Researchers require an understanding of task management and task complexity to ensure that the needs of caregivers are met in the application design^30–33^. Patient monitoring is also a popular feature, whereas different applications provided different types of monitoring parameters. These different types of patient monitoring functions may be useful for caregivers^34–36^. Education is another important feature that helps caregivers know what to expect with the patient’s disease progression and informs them about the available treatment options^24,27,29,37^.

One of the key findings in our review is the lack of comprehensive features (i.e., with multiple functionality categories) to support the caregivers. With each application providing only a few functionalities, caregivers may need to use multiple mHealth applications to fulfill all their needs. This may not meet the needs of caregivers and may also deter them from using the applications altogether. There is also inadequate mental health support for caregivers. Most importantly, the help for the early diagnosis of AD was not included as a feature for caregivers. On the other hand, many mHealth applications have been designed for AD patients to perform cognitive tests for AD diagnosis themselves. For example, some mHealth applications have been designed to evaluate and help enhance the cognitive skills of the patients through exercises and problems^22,24^. Our review can be used to guide the design and development of mHealth applications to provide comprehensive support for caregivers of AD patients.

The current standard of practice for AD diagnosis includes several cognitive tests, such as Saint Louis University Mental Status Examination (SLUM), Standardized Mini-Mental State Examination (SMMSE), and Montreal Cognitive Assessment Test (MoCA)^38–40^. Because the diagnosis of an AD patient relies heavily on the change in their cognitive abilities over time, these tests may need to be repeated multiple times. Having to visit the doctor’s office to perform these tests multiple times can frustrate the patient and subsequently affect the test performance. Repeating the same cognitive tests can also have a learning effect^38^. Both factors have been shown to decrease the sensitivity of the tests. These cognitive tests also lack specificity. Test results are usually given as an aggregate score and do not specify the patient’s detailed performance in each of the eight dimensions of cognition: visuospatial, naming, memory, attention, language, abstraction, delayed recall, and orientation^41^. These issues can be solved by integrating the cognitive tests into a mHealth application, which can also make administering these tests easier without compromising the integrity of the tests^42,43^.

As discussed earlier, caregivers are typically the first to detect changes in the patient’s cognitive skills and can be a great resource for the early diagnosis of AD patients. Thus, we recommend that mHealth application support for caregivers should include a functionality category of AD diagnosis tests. This functionality can include both the patient’s test performance in different cognitive dimensions and their cognitive impairment progression over time. The caregivers can utilize this functionality to not only administer the cognitive tests for early detection of cognitive impairment but also communicate the results with the patient’s physicians to support tailored treatment options^44^. We urge more research on how to digitize existing cognitive tests into mHealth applications for caregivers of AD patients with more active funding provided by the government and private organizations.

Although the benefits of incorporating AD caregivers into the patient’s care management are apparent, caregivers are often excluded from the patient care management or are not valued as part of the care team^45–47^. Many caregivers prefer to be actively involved with decision-making processes in the steps of AD care, from diagnosis to treatment^47^. For a disease characterized by progressive cognitive impairment, the information that caregivers can provide is invaluable. They can represent the patients and offer insight that no other healthcare professional in the care team can provide. Utilizing the caregivers as an asset within the healthcare team model can improve the care quality. Substitutable Medical Applications and Reusable Technologies (SMART) on Fast Healthcare Interoperability Resources (FHIR) provides an open-standards-based platform that allows a flow of information between clinical mobile applications and electronic health records (EHRs)^48^. SMART on FHIR can enable pertinent patient information sharing between healthcare professionals and caregivers through their mobile clinical applications^48,49^. FHIR makes it possible for caregivers to access EHR with appropriate security measures. In addition, technology platforms that enable the sharing of patient health information in compliance with the Health Insurance Portability and Accountability Act (HIPAA) provide grounds for developing and deploying secure mHealth applications^48,49^.

### Limitations

The absence of quality assessment of reviewed mHealth applications is a major limitation of this review, although it is not the focus of the study. There are no regulations or standards of quality assessment for mHealth applications, making it difficult to assess their quality^50^. For information-based content, such as education about the disease state, evaluation of the accuracy of the information presented is necessary. Thus, further research is needed to evaluate the quality of mHealth applications for caregivers of AD patients.

Furthermore, this review did not assess any caregiver’s opinions or engagement on the mobile applications. It is necessary to understand the need of caregivers to ensure the proper design of these applications. Future research should investigate if these mHealth applications reflect the demand of the targeted population while maintaining the quality and the accuracy of information. Finally, this review only examined mHealth applications through published academic literature. It also did not include any other digital tools independently available without smartphones. Feature research may include mHealth applications designed by practitioners or have not gone through a peer-review process.

## Conclusion

In this study, we reviewed literature consisting of 12 mHealth applications that provide support for caregivers of AD patients. We identified six themes of application functionalities, which are tracking, task management, monitoring, caregiver mental support, education, and caregiver communication platform. Among these features, tracking, task management, and monitoring were the most common. Our finding indicates that mHealth applications available for caregivers of AD patients are inadequate in terms of lack of comprehensive support, and the absence of functionalities for early AD diagnosis and the integration of caregivers in the patient’s care management. To accommodate the increasing number of caregivers due to the aging population, further research needs to expand caregivers’ roles in the care management of AD patients.

## Data Availability

No data sets were generated or analyzed during the current study. The aggregated data analyzed in this study are available from the corresponding author on reasonable request.
